# Transcranial magnetic stimulation in the treatment of adolescent depression: a systematic review and meta-analysis of aggregated and individual-patient data from uncontrolled studies

**DOI:** 10.1007/s00787-022-02021-7

**Published:** 2022-06-24

**Authors:** Christine Sigrist, Jasper Vöckel, Frank P. MacMaster, Faranak Farzan, Paul E. Croarkin, Cherrie Galletly, Michael Kaess, Stephan Bender, Julian Koenig

**Affiliations:** 1 Faculty of Medicine and University Hospital Cologne, Department of Child and Adolescent Psychiatry, Psychosomatics and Psychotherapy, University of Cologne, Cologne, Germany; 2 Department of Psychiatry, Cumming School of Medicine, University of Calgary, Calgary, AB Canada; 3 eBrain Lab, School of Mechatronic Systems Engineering, Simon Fraser University, Surrey, BC Canada; 4 Department of Psychiatry and Psychology, Mayo Clinic, Rochester, MN USA; 5 Adelaide Medical School, The University of Adelaide, Adelaide, SA Australia; 6 University Hospital of Child and Adolescent Psychiatry and Psychotherapy, University of Bern, Bern, Switzerland; 7 Department of Child and Adolescent Psychiatry, Centre for Psychosocial Medicine, University Hospital Heidelberg, Heidelberg, Germany; 8 Centre for Addiction and Mental Health, Department of Psychiatry, University of Toronto, Toronto, ON Canada; 9 Ramsay Health Care (SA) Mental Health, Adelaide, SA Australia; 10 Northern Adelaide Local Health Network, Adelaide, SA Australia

**Keywords:** Transcranial magnetic stimulation, Major depressive disorder, Adolescence, Meta-analysis, Individual patient data

## Abstract

Transcranial magnetic stimulation (TMS) is a non-invasive treatment for adolescent major depressive disorder (MDD). Existing evidence on the efficacy of TMS in adolescent MDD awaits quantitative synthesis. A systematic literature search was conducted, and data from eligible studies were synthesized using random-effects models. Treatment-covariate interactions were examined in exploratory analyses of individual-patient data (IPD). Systematic search of the literature yielded 1264 hits, of which 10 individual studies (2 randomized trials) were included for quantitative synthesis of mainly uncontrolled studies. Individual patient data (IPD) were available from five trials (all uncontrolled studies). Quantitative synthesis of aggregated data revealed a statistically significant negative overall standardized mean change (pooled SMCC = 2.04, 95% CI [1.46; 2.61], SE = 0.29, *p* < .001), as well as a significant overall treatment response rate (Transformed Proportion = 41.30%, 95% CI [31.03; 51.57], SE = 0.05; *p* < 0.001), considering data from baseline to post-treatment. Exploratory IPD analyses suggests TMS might be more effective in younger individuals and individuals with more severe depression, and efficacy might be enhanced with certain treatment modality settings, including higher number of TMS sessions, longer treatment durations, and unilateral and not bilateral stimulation. Existing studies exhibit methodological shortcomings, including small-study effects and lack of control group, blinding, and randomization—compromising the credibility of the present results. To date, two randomized controlled trials on TMS in adolescent depression have been published, and the only large-scale randomized trial suggests TMS is not more effective than sham stimulation. Future large-scale, randomized, and sham-controlled trials are warranted. Future trials should ensure appropriate selection of patients for TMS treatment and guide precision medicine approaches for stimulation protocols.

**Supplementary Information:**

The online version contains supplementary material available at 10.1007/s00787-022-02021-7.

## Introduction

Adolescent major depressive disorder (MDD) presents a serious and oftentimes life-threatening disorder, with the potential to disrupt normal development, and to impede the quality of life of affected individuals and their families [1, 2]. It has been recognized that MDD is a leading contributor to the burden of disease in young individuals aged 10–24 years [3], yet, currently available options for the treatment of MDD in adolescents remain unsatisfactory. In the past 2 decades, pharmacological and psychological interventions have been widely used in the treatment of MDD in adolescents. Compared to other psychiatric disorders in young individuals, such as anxiety, attention-deficit hyperactivity disorder, or conduct-related problems and disorders, mean effects for the treatment of MDD are, however, modest [4]. Antidepressants, except for fluoxetine, may not offer a clear advantage over placebo for a large percentage of individuals [5, 6]. Of concern, one-third of MDD patients who undergo treatment do not achieve remission after having gone through various treatment options, which can lead to treatment-resistant depression (TRD; [7–9]).

Increasingly, transcranial magnetic stimulation (TMS) has been used to both study and treat neuropsychiatric and neurological disorders. TMS is a technique employed for transcranial stimulation of the brain using a magnetic coil positioned on the surface of the head usually tangential to the scalp, and is based on the principle of electromagnetic induction [10]. A brief, high-current pulse is produced in the magnetic coil, resulting in a magnetic field, passing perpendicularly to the plane of the magnetic coil, inducing an electric field perpendicularly to the magnetic field on the surface of the cortex, depolarizing neurons or their axons [11, 12]. Repetitive TMS (rTMS) involves modalities to deliver multiple pulses of stimulation in a short interval of time and at various stimulation frequencies (e.g., 1, 5, or 10 Hz). As compared to single-pulse or paired-pulse TMS protocols, rTMS produces longer-lasting changes in neural activity [13] and is most commonly used in clinical settings. rTMS has been suggested to modulate brain network functioning [14, 15]. Theta burst stimulation (TBS) involves the application of 50 Hz bursts at theta (5 Hz) frequency. This patterned stimulation can deliver a high number of pulses and is thought to confer neurophysiological and therapeutic effects in a shorter time as compared to standard TMS (i.e., in about 40 s to 3 min compared to about 30 min in, e.g., 10 Hz rTMS [16, 17]). The intensity of TMS is generally determined relative to the resting motor threshold (RMT) of the individual patient. RMT is assessed via the primary motor cortex and serves as a proxy for the activation of other cortical regions [18, 19]. Typically, TMS is applied with intensities ranging from 80 to 120% of RMT, and the efficacy of stimulation (besides the state of the receiving brain) generally strongly depends on the stimulation protocol (i.e., dose) [20, 21]. Magnetic stimulators equipped with figure-eight coils have obtained regulatory approval for clinical use in many countries, and are most commonly used in clinical therapeutic settings. Figure-eight coils induce electric fields in the target area with greater focality compared to coil types shaped differently (i.e., concentric coils), also minimizing the potential risk of side effects caused by stimulation of surrounding areas (for a discussion of geometric variations of figure-eight coils and implications for therapeutic use, see, e.g., [22]).

In 2008, the United States Food and Drug Administration (FDA) cleared the first TMS device for therapeutic clinical use in adult MDD [23, 24]. To date, multiple meta-analyses have demonstrated that TMS applied to the dorsolateral prefrontal cortex (DLPFC) is effective, specifically for cases of TRD [25–27]. In a number of existing reviews, TMS has also been ascribed potential as a safe and effective treatment for MDD in adolescents [28–32]. However, findings thus far have been mixed, and quantitative syntheses are currently lacking on TMS in adolescent MDD. Thus, it remains unclear under what conditions and to what extent TMS is effective for the treatment of MDD in youth.

During the neurodevelopmental period of adolescence, aberrations from normative neuro-maturational processes potentially underlie the pathophysiology of depression [33]. Specifically, drastic changes in structural and functional brain architecture may lead to imbalances in excitation and inhibition, changes in cortical plasticity and connectivity, and less effective information exchange between brain regions that are critical to the processing of emotion [34–36]. Of note, it has been suggested previously that neurodevelopmental processes in adolescence might contribute to the currently inconsistent results between studies investigating rTMS in adolescent MDD [37].

A range of further patient-related factors are likely to influence how rTMS is received and processed in the brains of adolescents and adults alike, and in turn, the influence of individual-patient factors on TMS treatment outcome needs to be investigated in adolescent samples. Of note, meta-analytic evidence in adults suggests *age* to present one of the most important predictors of TMS treatment response, with young age presenting a good prognostic factor [38–40]—which is also in line with psychotherapeutic and psychopharmacological treatment studies in children and adolescents, suggesting younger patients to be more likely to respond to treatment [41, 42]. Besides patient demographics, stimulation-parameter settings are likely to influence effect size. In a previous meta-analysis of adult patients, stimulation intensity, frequency, and site of stimulation, as well as the course of treatment, were identified as moderators of the treatment outcome [43]. Furthermore, the efficacy of TMS might strongly depend on accurate targeting of the region to be stimulated, while localization of the cortical target region currently lacks standardization. In many, and particularly in earlier studies, the DLPFC as a target site has been approximated from measurements on the scalp, using the so-called “5-cm rule” involving measurement to a location about 5 cm anterior to the Motor Threshold location in the anterior–posterior plane [44, 45]. A further scalp-based targeting method that has found wide application is the Beam F3 method, which additionally accounts for head size and shape [46]. More recently, studies have started to use structural or functional magnetic resonance imaging (MRI) and/or diffusion tensor imaging (DTI) combined with neuro-navigation systems to target specific regions of interest [47–49]. Critically, targeting of specific brain regions by imaging and neuro-navigation seems to result in larger effect sizes as compared to scalp-based approaches [49–51].

Especially in more recent years, consensus has been reached that TMS might be a valuable treatment option for MDD in youth. This same conclusion has been drawn repeatedly from a number of existing systematic literature reviews on the topic ([28–32, 37, 52]), two of which [31, 52] also include a systematic assessment of study quality and risk of bias, respectively. Crucially, among the currently existing studies examining efficacy of TMS treatment in adolescent depression, there are only two randomized, double-blind sham-controlled trials [53, 54]—and, what is more, the largest and as of yet best-designed study [54] found no additive effect of rTMS compared to sham stimulation considering the reduction of depressive symptoms in adolescents. Most probably based on the current lack of large-scale, high-quality, randomized studies, a quantitative synthesis of the existing evidence on the efficacy of TMS treatment in adolescent MDD is lacking from the literature, and potential moderators of treatment outcome have not yet been meta-analytically examined.

Thus, to tackle these respective gaps, the current systematic review and meta-analysis aims to first summarize the currently existing data on efficacy (defined as pre- to post-treatment change and response rate) of TMS treatment in adolescent MDD on a study level, and second, in exploratory analyses on the level of individual-patient data (IPD), to examine patient- and treatment-related factors which potentially influence the efficacy of TMS in adolescent MDD.

## Methods

The present study protocol was pre-registered through a web-based protocol on the International Prospective Register of Systematic Reviews (PROSPERO; e.g., [55]), available from https://www.crd.york.ac.uk/prospero/display_record.php?ID=CRD42020210008. Updates to the current review will be posted to the protocol. Throughout the meta-analytic process, we followed the current recommendations from the Preferred Reporting Items for Systematic Reviews and Meta-Analyses statements (PRISMA; PRISMA-IPD; [56, 57]), and consulted the Cochrane Handbook for Systematic Reviews of Interventions providing gold-standard advice in conducting systematic reviews on the effects of healthcare interventions [58].

### Search strategy

For the identification of studies investigating TMS in adolescent depression, a systematic search was conducted, drawing on the following databases: PubMed, PsycINFO and Web of Science with the search terms (TMS OR transcranial magnetic stimul* OR TBS OR theta burst stimul*) AND (child* OR teen* OR adolescen* OR kid* OR juvenile* OR pediatric OR early onset OR early-onset OR youth) AND (depress* OR MDD OR mood OR affect* OR dysthym*). Publications up to 30 October 2020 were included in the search. In addition, we searched the bibliographies of published reviews on TMS treatment in adolescent depression [29–59]. A systematic search of the clinical trial registers of the U.S. National Library of Medicine (ClinicTrials.gov) and the World Health Organization (International Clinical Trials Registry Platform) was conducted to identify potential unpublished trials. The authors of primary studies were contacted to obtain unpublished data.

### Study selection

Article screening was performed using the Rayyan screening software [60], which allowed transparent documentation on decisions concerning included and excluded studies. Any uncertainty over the eligibility of particular studies was resolved through discussion of the review authors until agreement was reached. Based on titles and abstracts screening, studies meeting the following criteria were included: (1) studies investigating TMS or TBS treatment in (2) adolescent samples with a mean age range of 12–21 years (3) with a clinical diagnosis of depression, (4) written in English or German language. We endorsed no restrictions on the study design and included controlled or uncontrolled multi-subject trials as well as case reports. Of note, we included any study irrespective of statistical significance of reported treatment effects (i.e., no selection criteria were applied with regard to statistical significance of study results). We included journal articles, conference abstracts and letters to the editor. Studies were excluded if they met the following criteria: (1) studies investigating non-human subjects (e.g., rats or mice), (2) studies only investigating adults, (3) studies using neuro-stimulation techniques different from TMS or TBS (e.g., transcranial direct current stimulation or electroconvulsive therapy), (4) studies investigating clinical diagnoses other than depression (e.g., epilepsy, cerebral palsy, schizophrenia, autism, Tourette syndrome, and attention-deficit hyperactivity disorder), and (5) studies where the severity of depressive symptoms had not been assessed using standardized measures. In addition, published study protocols and clinical trial registrations—not reporting empirical data—were excluded from further synthesis.

### Primary and secondary outcome measures

Our primary aim was to synthesize existing evidence on the efficacy of TMS in adolescent depression and to identify, in an exploratory approach, respective moderators on both the study and individual-patient levels. Based on the population of primary studies available, efficacy, on the study level, was defined as standardized mean change (SMCC) between pre- and post-treatment assessments, considering the severity of depressive symptoms. Furthermore, we considered response rate, measured as the raw percentage of responders at treatment termination, as a secondary outcome measure of efficacy. Response rate was defined as the percentage of individuals within a study sample in which a reduction of at least 50% in depressive symptoms from baseline to treatment termination was observed. At the level of individual patients, we examined patient- and treatment-level characteristics as potential moderators of pre- to post-treatment change as well as treatment response (as described in further detail in statistical analysis below).

### *Data extraction and preparation*

To obtain aggregated data on the study level, the following information was systematically extracted from each included study in tabular format: (1) authors, publication year, title, and name of the journal where the study had been published, (2) aims and objectives of the study, (3) study design, (4) sample characteristics (e.g., sample size, sex distribution of the sample, mean age and age range, diagnosis, any other relevant sociodemographic and clinical characteristics), (5) methodological aspects (e.g., treatment realization, measurement of variables, and conduction of analysis), (6) stimulation protocol (i.e., coil location, number of TMS sessions, number of pulses per session, session duration, TMS frequency and intensity, (7) main outcomes (i.e., depression severity and response rates), (8) additional outcomes (i.e., adverse events), and (9) potential strengths of the study and its limitations. Missingness was recorded, and following data extraction, the study authors were contacted to gather missing data on the trials. In case data were presented for subgroups of the sample only (e.g., presenting data for responders vs. non-responders), the respective means and SDs were combined by decomposing the mean and SD (Higgins et al., 2021). To obtain IPD, we sent data requests to all corresponding study authors (e.g., we gathered patient ID, sex, age, and severity of depressive symptoms at pre- and post-treatments, as well as information on applied stimulation protocols, such as frequency or intensity). If the study authors could not be reached via data requests, IPD was extracted from published articles, if available. We combined IPD into a single, consistent data set, while any data issues (e.g., invalid, or out-of-range scores) were resolved in collaboration with the primary study authors, if manageable. Data preparation was conducted using Stata/SE version 17.0 (StataCorp, 2019).

### Statistical analyses

We first conducted a meta-analysis of aggregated data gathered from primary studies addressing the efficacy of TMS treatment in adolescent depression. Two separate meta-analyses were conducted, considering Standardized Mean Change Score (SMCC) and response rate, respectively. Second, IPD meta-analysis, contemporaneously regarded as the gold standard of meta-analysis, was conducted on a subsample of studies for which IPD were available. Again, two different types of analyses were conducted, considering treatment efficacy as well as response. IPD meta-analysis allowed us to examine potential treatment interactions with the patient- and study-level characteristics.

### Meta-analysis of aggregated data

To conduct a meta-analysis of existing studies on the efficacy of TMS treatment in adolescent depression, based on aggregated data from each primary study, we calculated SMCC and the accompanying 95% confidence interval (CI) between pre- and post-treatment depression scores. To calculate SMCC, information on total sample size, pre- and post-treatment means, standard deviations, and the strength of association (correlation coefficient) between pre- and post-treatment values are required [61, 62]. Thus, if the correlation coefficient between pre- and post-treatment scores had not been reported and could not be calculated directly over IPD data, as was the case for many studies, the respective correlation was estimated over available summary statistics (e.g., pre- and post-treatment means and SD, SMD). Individual effect size estimates from primary studies were subsequently pooled using a random-effects model [63]. We used restricted maximum likelihood estimation (REML) where effect size and heterogeneity estimates are achieved iteratively, as generally recommended [64, 65]. Heterogeneity between primary studies was tested for significance using the Cochrane *Q* statistic, testing the null hypothesis that all variation in effect size estimates across studies is due to sampling error [66]. The amount of true heterogeneity across studies was further estimated via the *I*^*2*^ statistic, indexing the proportion of heterogeneity across studies not due to random error. Following widely used conventions [67], we interpreted an *I*^*2*^ statistic of 25% as small, 50% as moderate, and 75% as to signify high levels of heterogeneity. We next applied the conventional Egger’s regression test [68, 69] to explore the likely presence (Egger’s regression test: *p* < 0.1) or absence (Egger’s regression test: *p* ≥ 0.1) of small-study effects [70]. Furthermore, different variants of the funnel plot as a diagnostic tool widely applied in meta-analysis were created. Specifically, we created traditional funnel plots, which allowed us to examine whether smaller studies inherent of larger standard errors and associated lower analytic power tended to yield larger ES (examination of small-study effect): scatter plots of the effect size estimates from primary studies against their standard errors were plotted, including a funnel centered on the summary effect size [68, 71]. Then, to more closely examine the distribution of studies with regard to statistical significance, we also created contour-enhanced funnel plots centered on zero, including contours that represent conventional levels of statistical significance (i.e., ≤ 0.01, ≤ 0.05, ≤ 0.10, and ≤ 1.00) as a graphical aid for interpretation [72]. Finally, in addition to summarizing effect sizes over all primary studies, we conducted sensitivity analyses in the form of subgroup meta-analysis: first, we summarized a subgroup of studies based on applied frequency and target location (i.e., including only studies where high-frequency stimulation was applied unilaterally to the left DLPFC), secondly, subgroups of studies were summarized based on the method used to localize the target region (i.e., studies using scalp-based methods vs. neuro-navigation techniques), and third, subgroups of studies were summarized based on study design (i.e., randomized sham-controlled trials vs. uncontrolled studies).

To obtain a measure of aggregated response rate, for each primary study, the absolute number of responders in each study sample was converted to a measure of raw proportions. Measures of the raw proportion of treatment responders obtained for each primary study (as available) were further synthesized using a random-effects model and REML, resulting in the summary estimate of Transformed Proportion [62]. Model diagnostics, heterogeneity measures, and sensitivity analyses (subgroup meta-analysis) were applied as described above.

### *Meta-analysis of individual-patient data*

Following traditional meta-analysis, we performed exploratory one-stage IPD meta-analysis, exploring treatment effects on the patient-level and in interaction with patient- as well as treatment-level characteristics. One-stage models might be particularly suitable if only a few or small studies are subjected to IPD meta-analysis, as they generally improve the statistical power to detect treatment by covariate interactions [57, 73, 74]. With regards to our primary outcome, we used mixed-effects linear regression, considering separately each depression scale deployed in at least two independent primary studies for which IPD were available (which included the Hamilton Depression Rating Scale, HDRS; Beck Depression Inventory-II, BDI-II; and the revised version of the Children’s Depression Rating Scale, CDRS-R). In the respective models, accounting for clustering of measurements within individuals clustered within trials, we explored pre-treatment to post-treatment change in depression scores (commonly used formula: outcome ~ 1 + time + (1 | lab) + (1 | participant)). Furthermore, we examined treatment interactions with the patient-level characteristics age (in years), sex (0, male and 1, female), and pre-treatment (baseline) depression scores, and with the trial-level characteristics ‘laterality’ (unilateral or bilateral stimulation applied), total number of sessions, treatment duration (in days), and the TMS modality applied (standard rTMS or TBS). Each of these moderators or treatment-covariate interactions were tested one at a time (commonly used formula: outcome ~ 1 + time * covariate + (1 | lab) + (1 | participant)). Considering treatment response (0, 1; binary), mixed-effects logistic regression models, accounting for clustering of individuals within trials, were computed, while we examined the potential influence of the above patient- and trial-level covariates in predicting treatment response vs. non-response, again considering each covariate at a time (commonly used formula: response ~ 1 + covariate + (1 | lab)). Of note, given the exploratory nature of these secondary analyses, no corrections for multiple comparisons were applied.

## Results

### Search results

**Fig. 1:**
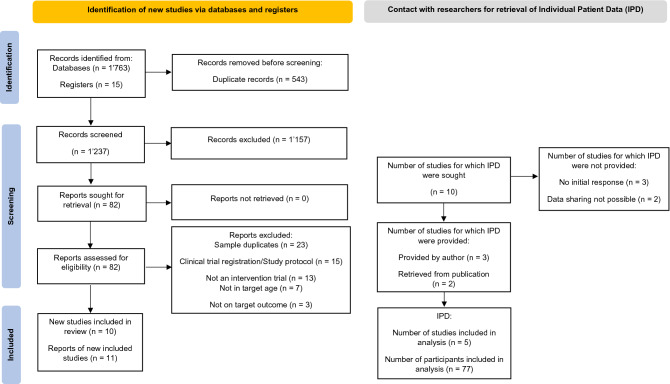
Study selection process according to the PRISMA and PRISMA-IPD flow diagram (Stewart et al. 2015; Page et al. 2020)

In Fig. 1, the study selection process is illustrated based on current versions of the PRISMA and PRISMA-IPD flow diagrams [56, 57]. After the identification of potentially eligible studies, all duplicates were removed. We screened a total of 1237 titles and abstracts from original articles, resulting in 82 studies forwarded to full-text assessment, including records from additional sources. From the initial pool of potentially eligible studies, 11 studies were retained for qualitative synthesis. All corresponding study authors were contacted to retrieve further information on the respective data, and to gather IPD. During this stage, one study [75] was excluded because the respective sample entirely overlapped with the sample from a more recent publication with a larger sample size [76]. As follows, *N* = 10 individual study samples were subjected to the quantitative synthesis of aggregated data [53, 54, 76]–[82]. Upon contacting the study authors of all primary studies, IPD was provided for three studies via the author correspondence [48, 76, 78]. Moreover, for two studies, IPD could be extracted from published research articles [77, 79]. Considering the remaining studies, IPD could not be retrieved because either, the corresponding authors did not respond to our initial as well as several repeated follow-up data requests (*n* = 3; [53, 81, 82]), or because the respective study data could not be shared due to industry sponsorship and respective regulations (*n* = 2; [54, 80]).

### Included studies

In Table 1, summary characteristics of each study included in the present quantitative analyses are presented. In total, the 10 studies identified included *N* = 247 participants, with a mean number of *N* = 25 (SD = 15) participants per study, a mean age of 17.45 (mean SD = 1.99) years, and on average, samples included 63.47% females. Sample sizes ranged from *N* = 8 to *N* = 48 participants. Of all studies included, two reported on the ethnicity of their included sample. Three studies had been conducted in China, three studies in the US, two in Canada, one in Australia, and one study had been conducted in Israel. Considering study design, two studies adopted a double-blind, randomized, and sham-controlled design, while the remaining eight studies were open-label studies, without a control group. Considering primary outcome measures, the number of deployed depression scales ranged from one to five (see further Table 1). Participants in all studies were under antidepressant treatment while receiving TMS, including antidepressant medication alone (seven studies) or a combination of antidepressants and psychotherapy (three studies; please see Table 1). Considering TMS treatment regimen, nine studies applied standard rTMS, and in one study, a combination of continuous and intermittent TBS (cTBS and iTBS) was used. Eight studies applied unilateral stimulation, of which six targeted the left DLPFC, one stimulated the left PFC, and one study targeted either the left or the right DLPFC. Of the remaining two studies, one applied bilateral stimulation, targeting both the left and right DLPFC, and in one study, either the right DLPFC was stimulated or stimulation was applied bilaterally (left and right DLPFC). Four studies localized the target region(s) based on a neuro-navigation technique, while five studies applied scalp-based localization using the 5-cm-rule (in one study, this was 6 cm). One study did not report on the localization of the target region. Mean treatment duration was 3.5 weeks (SD = 1.73; range: 1–6), and on average, treatment sessions lasted for 35 min (SD = 7.42; range: 20–40). Considering stimulation-parameter settings, number of pulses per session ranged from 1800 to 6000, with stimulation of 2–5 s per train and 12–58 s per interval. Frequency was reported at 10 Hz except for TBS at 50 Hz (one study), and stimulation intensities ranged from 80 to 120 RMT (one study used the ‘active motor threshold’; AMT).

**Table 1:** Summary characteristics of primary studies included in the present meta-analysis (*N* = 10), investigating TMS for treatment of adolescent depression (ordering by year of publication)

Study	Sample size (*N*)	Sample characteristics	Adjunctive treatment	Age (range, mean ± SD)	TMS location; targeting	No. TMS sessions	No. pulses per session	Session duration (min); sec./train; sec./interval	TMS freq. (Hz)	Intensity (%)	Outcome measure	Reported pre–post-mean change (SD)	Pre–post-SMCC (Var.)	Number of responder (≥ 50% reduction)	Included in IPD meta-analysis
Pan et al. [22]	42 (21 sham)	Treatment naïve patients with MDD	Antidepressant medication	14–28, active: 18.14 ± 3.94, sham: 21.43 ± 6.79	Left DLPFC	7	6000	n.a 5/15	10	100 (RMT)	HDRS-24, MADRS	Active^a^: – 19.19 (8.72) (sig.); Sham^a^: – 4.48 (6.27) (n.s.)	Active^a^: 2.12 (.15); Sham^a^: .69 (0.05)	*n*.*a*	No
Zhang et al. [81]	42	Depression with comorbid anxiety symptoms	Antidepressant medication	13–16, 15.24 ± 1.61	Right or left DLPFC	20	2400	n.a n.a./12	1 or 10	80–120 (RMT)	HDRS-17	n.a (sig.)	3.09 (.14)	n.a	No
Croar-kin et al. [54]	103 (55 sham)	Meeting DSM-IV criteria for diagnosis of unipolar MDD and current episode; TRD, defined as an antidepressant treatment record level of 1 to 4 in a current episode of depression	Antidepressant medication	12–21, active: 17.6 ± 2.28, sham: 17.1 ± 2.22	Left PFC	30	3000	37 4/26	10	110–120 (RMT)	HDRS-24, HDRS-17, MADRS, CDRS-R, QIDS-A_17_-SR	Active^a,b^: – 11.1 (2.03) (sig.); Sham^a,b^: – 10.6 (2.00) (sig.)	Active^a^: 1.08 (.03); Sham: .65 (0.02)	Active: 20/48 (41.7%); Sham: 20/50 (36.4%)	No
Dhami et al. [48]	20	Diagnosis of depression; eligible if not gone through any changes during treatment (psychotherapy and medication) for at least 4 weeks prior to the study	Antidepressant medication; psychotherapy	16–24, 20.90 ± 2.60	Right and left DLPFC (bilateral); neuro-navigation	10	1800 left and 1800 right	n.a n.a./n.a	50 (cTBS and iTBS)	80 (AMT)	HDRS-17; BDI-II, CDRS-R	n.a (sig.)	2.22 (.18)	4/20 (20%)	Yes
Rose-nich et al. [78]	15	Adolescents and young adults with major depression episode	Antidepressant medication; psychotherapy	17–25, 20.69 ± 2.55	Right unilateral or bilateral DLPFC	18	900–2400	15 or 30 5/25	1 or/and 10	110	HDRS-17, MADRS, Zung	– 7.23 (n.a.)^a^ (sig.)	1.22 (.12)	6/15 (40%)	Yes
Mac-Master et al. [76]	32	Diagnosis of MDD, based on K-SADS-PL, with a symptom severity of 40 or greater on the CDRS-R, and a history of failing to respond to at least one SSRI trial (minimum 8-week treatment at an adequate dose; retrospectively determined via interview)	Antidepressant medication	13–21, 17.57 ± 1.98	Left DLPFC; neuro-navigation	15	3000	37.5 4/26	10	120	HDRS, CDRS-R, BDI-II	– 10.88 (6.87)^a^ (sig.)	1.54 (.07)	18/32 (56%)	Yes
Zhang et al. [82]	42	Meeting criteria for mood or anxiety disorders defined in the DSM-IV, experienced an acute exacerbation of the symptoms of depression or anxiety, or had a baseline score of at least 14 points on the 17-items on the HDRS or at least 10 points on the 14-items HAMA	Antidepressant medication	10–17, 14.6 ± 2.0	Left DLPFC	20	2400	*n*.*a* *n*.*a*./12	10	120	HDRS-17, HAMA-14	n.a (sig.)	3.37 (.16)	15/15 (100%)	No
Wall et al. [80]	10	Active treatment for MDD based on DSM-IV; at least one prior failed antidepressant medication trial	Antidepressant medication	13- 17, 15.9 ± 1.1	Left DLPFC; neuro-navigation	30	3000	37.5 4/26	10	80–120	CDRS-R, QIDS-A_17_-SR	n.a (sig.)	2.01 (.30)	6/10 (60%)	No
Wall et al. [79]	8	Adolescents with MDD; non-response to 2 adequate antidepressant trials (i.e., treated with stable SSRI dose regimen for at least 6 weeks)	Antidepressant medication	14–17, 16.54 ± 1.18	Left DLPFC	30	3000	37.5 4/26	10	120	CDRS-R, QIDS-A_17_-SR	– 33.29 (7.39)^c^ (sig.)	4.01 (1.13)	n.a	Yes
Bloch et al. [77]	9	MDD based on DSM-IV diagnostic criteria; resistant MDD defined as failure of at least 1 course of psychotherapy and 2 courses of medications over 8 weeks each, at least one of them with fluoxetine (initially 20 mg/d and later 40 mg/d)	Antidepressant medication; psychotherapy	16–18, 17.30 ± 0.62	Left DLPFC	14	*n*.*a*	*n*.*a* 2/58	10	80	CDRS-R, BDI-II	– 16.67 (14.64)^c^ (sig.)	1.02 (.17)	n.a*.* (response rate defined as 30% reduction of CDRS-R score)	Yes

*AMT* Active motor threshold, *BDI-II* Beck Depression Inventory-II, *CDRS-R* Children’s Depression Rating Scale–Revised, *DLPFC* dorsolateral prefrontal cortex, *HAMA* Hamilton Anxiety Rating Scale, *HDRS-17* 17-item Hamilton Depression Rating Scale, *HDRS-24* 24-item Hamilton Depression Rating Scale, *K-SADS-PL* Schedule for Affective Disorders and Schizophrenia for School-Age Children-Present and Lifetime Version, *MADRS* Montgomery–Asberg Depression Rating Scale, *MDD* major depressive disorder, *SSRI* selective serotonin reuptake inhibitors, *QIDS-A*_*17*_*-SR* Quick Inventory of Depressive Symptomatology‒Adolescent (17-Item)‒Self-Report, *RMT* resting motor threshold, *TRD* treatment-resistant depression, *Zung* Zung Self-Rating Depression Scale

^a^HDRS score

^b^Least-squares

^c^CDRS score

### Meta-analyses on aggregated data

#### Pre- to post-treatment change

**Fig. 2:**
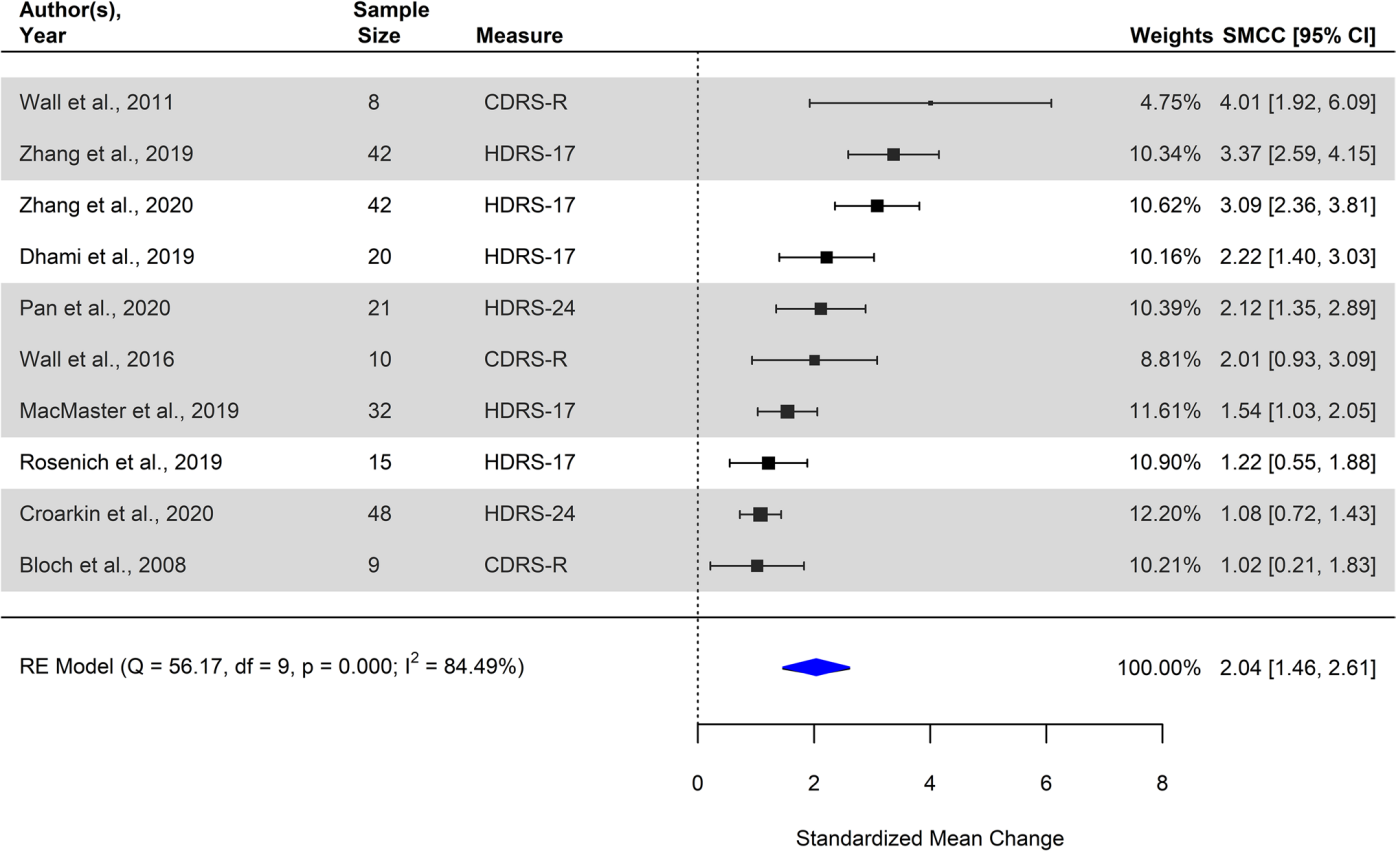
Forest plot of individual observed estimates of standardized mean change (SMCC) including corresponding weights, 95% confidence intervals and the pooled summary model using random-effects, examining the pre- to post-treatment change in depression scores. Studies with high-frequency stimulation applied unilaterally to the left DLPFC are highlighted in gray. *CDRS-R* Revised version of the Children’s Depression Rating Scale, *HDRS-17/24* Hamilton Depression Rating Scale, 17-item/24-item version

Synthesis of all 10 studies examining pre- to post-treatment change in depression scores resulted in a significant pooled effect size estimate (pooled SMCC = 2.04, 95% CI [1.46; 2.61], SE = 0.29, *p* < 0.001), implying a statistically significant reduction in depression scores measured at post-treatment compared to respective pre-treatment (baseline) scores. Individual and summary effect size estimates as observed are depicted in a forest plot (Fig. 2). The resulting Chi-square *Q* statistic exceeded the level of statistical significance, suggesting significant between-study heterogeneity (*Q*_10_ = 56.17, *p* < 0.001). The estimate *I*^2^ suggested that 84.49% (95% CI [65.76–96.20]) of the variance in estimates of effect size was due to true variation between primary studies rather than mere sampling error, conventionally regarded as a large amount of heterogeneity between studies.

**Fig. 3:**
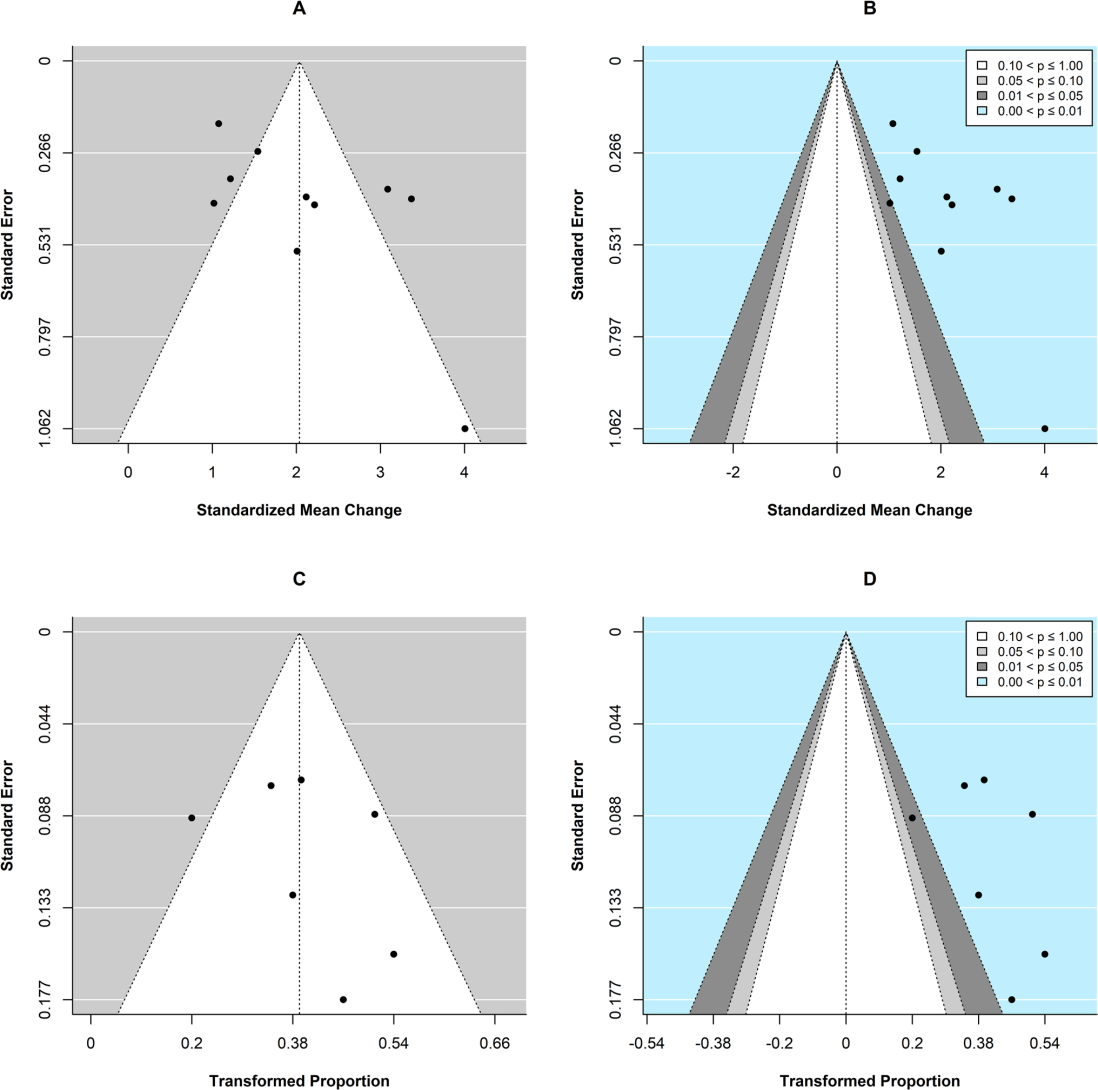
Funnel plots of individual observed effect size estimates of standardized mean change (SMCC) between pre- to post-treatment depression scores against the corresponding standard errors (i.e., the square root of the sampling variances) on the y-axis, and of Transformed Proportion of treatment responders against the corresponding standard errors on the y-axis, to aid assessment of potential small-study bias. **A**, **C**: Traditional funnel plots centered at the observed summary effect (SMCC and Transformed Proportion, respectively). **B**, **D**: Contour-enhanced funnel plots centered at zero including grey-shaded regions that indicate various levels of statistical significance: the unshaded region in the middle of the funnel corresponds to *p* values greater than 0.10, the dark grey-shaded region corresponds to *p* values between 0.10 and 0.05, the light grey-shaded region corresponds to *p* values between 0.05 and 0.01, and the region outside of the funnel (light blue) corresponds to *p* values below 0.01 (group comparison and correlational meta-analysis, respectively)

The corresponding funnel plots of individual observed estimates of effect size against their standard error (A) and the respective contour-enhanced version (B) are shown in Fig. 3. Visual inspection of the funnel plots A and B (Fig. 3) suggested likely presence of small-study bias, which was in accordance with the results from Egger’s regression test of funnel plot asymmetry (*z* = 2.06, *p* = 0.039). Furthermore, five studies seemed to be outliers as by visual inspection of funnel plot A (Fig. 3), and in all but one study, which fell in the range of *p* values between 0.01 and 0.05, *p* values were located in the range between 0.001 and 0.01.

Considering sensitivity analyses, synthesis of a subgroup of seven studies applying high-frequency stimulation solely to the left DLPFC (as opposed to the remaining three studies where high- and/or low-frequency stimulation was applied uni- and/or bilaterally) resulted in an overall significant pooled effect size estimate (pooled SMCC = 1.99, 95% CI [1.26; 2.72], SE = 0.37, *p* < 0.001), suggesting a statistically significant reduction in depression scores. Second, syntheses of two subgroups of studies based on localization of the target region resulted in an overall significant pooled effect size estimate for the subgroup of studies where a scalp-based method had been used (*k* = 5, pooled SMCC = 1.97, 95% CI [0.83; 3.10], SE = 0.58, *p* < 0.001), as well as the studies where a neuro-navigation technique had been applied (*k* = 4, pooled SMCC = 1.87, 95% CI [1.47; 2.28], SE = 0.20, *p* < 0.001). Third, syntheses of two subgroups of studies based on study design resulted in an overall significant pooled effect size estimate for the subgroup of randomized controlled trials (*k* = 2, pooled SMCC = 1.54, 95% CI [0.53; 2.55], SE = 0.52, *p* = 0.003), as well as the subgroup of uncontrolled studies (*k* = 8, pooled SMCC = 2.18, 95% CI [1.50; 2.86], SE = 0.34, *p* < 0.001).

#### Response rate

**Fig. 4:**
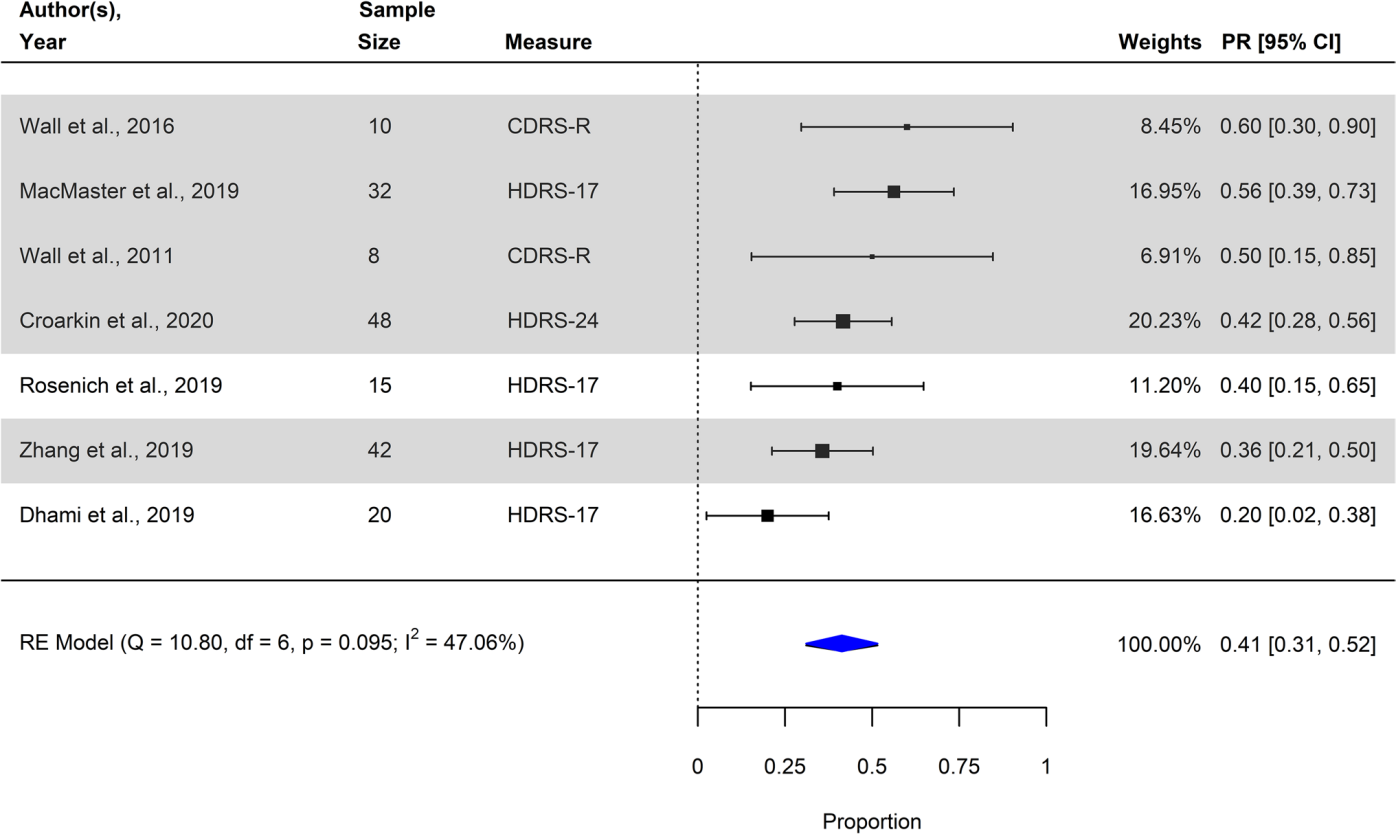
Forest plot of individual observed estimates of raw and transformed proportion of responders including corresponding weights, 95% confidence intervals and the pooled summary model using random-effects. Studies with high-frequency stimulation applied unilaterally to the left DLPFC are highlighted in gray. *CDRS-R* Revised version of the Children’s Depression Rating Scale; *HDRS-17/24* Hamilton Depression Rating Scale, 17-item/24-item version

By pooling seven individual observed estimates of the raw proportion of treatment responders, we found a significant summary estimate (Transformed Proportion: 0.41 (41.30%), 95% CI [0.31; 0.52], SE = 0.05; *p* < 0.001), implying a statistically significant treatment response rate. A forest plot depicting individual and summary estimates of response rate is illustrated in Fig. 4. The Chi-square *Q* statistic was non-significant (*Q*_7_ = 10.80, *p* = 0.095), suggesting that variation in ES estimates might be due to sampling error rather than true between-study heterogeneity. Nonetheless, the estimate *I*^2^ suggested 47.06% (95% CI [0.00; 88.94]) of the variance in estimates of effect size to be due to true variation between primary studies rather than mere sampling error, conventionally regarded as a moderate amount of heterogeneity between the studies.

Visual inspection of the funnel plots C and D (Fig. 3), and in accordance with the results from Egger’s regression test of funnel plot asymmetry (*z* = 0.93, *p* = 0.354), did not clearly indicate the presence of publication bias. One study was identified as outlier as by visual inspection of funnel plot C (Fig. 3). Again, in all but one study, which fell in the range of *p* values between 0.01 and 0.05, *p* values were located in the range between 0.001 and 0.01. Considering sensitivity analyses, synthesis of a subgroup of 5 studies applying high-frequency stimulation solely to the left DLPFC (as opposed to the three remaining studies where high- and/or low-frequency stimulation was applied uni- and/or bilaterally) resulted in an overall significant pooled effect size estimate (Transformed Proportion: 0.46 (45.52%), 95% CI [0.36; 0.55], SE = .0.05, *p* < 0.001) suggesting a statistically significant treatment response rate. Second, syntheses of two subgroups of studies based on localization of the target region resulted in overall significant pooled effect size estimates for both the subgroup of studies where a scalp-based method had been used (*k* = 4, Transformed Proportion: 0.40 (39.72%), 95% CI [0.31; 0.49], SE = 0.05, *p* < 0.001) and the studies where neuro-navigation had been applied (*k* = 3, Transformed Proportion: 0.44 (44.12%), 95% CI [0.18; 0.70], SE = 0.13, *p* < 0.001). Third, syntheses of two subgroups of studies based on study design resulted in an overall significant effect size estimate for the subgroup of randomized controlled trials (*k* = 1, Transformed Proportion = 0.42, 95% CI [0.28; 56], SE = 0.07, *p* =  < 0.001), as well as the subgroup of uncontrolled studies (*k* = 6, Transformed Proportion = 0.42, 95% CI [0.29; 54], SE = 0.07, *p* =  < 0.001).

### Meta-analyses on individual-patient data (one-stage models)

In total, *N* = 5 independent datasets from uncontrolled studies were subjected to IPD meta-analysis. Data included various depression scales. We conducted IPD meta-analysis individually for each depression scale, if at least two independent datasets included the respective scale as primary outcome measure. Accordingly, we conducted separate analyses including data on the HDRS (as provided for 3 studies, *N* = 61), CDRS-R (provided for 4 studies, *N* = 51), and BDI-II (provided for 3 studies, *N* = 61). Correlation coefficients between pre- and post-treatment scores from all three measures are provided in Table S1 in the Supplementary Materials.

#### Pre- to post-treatment change

**Fig. 5:**
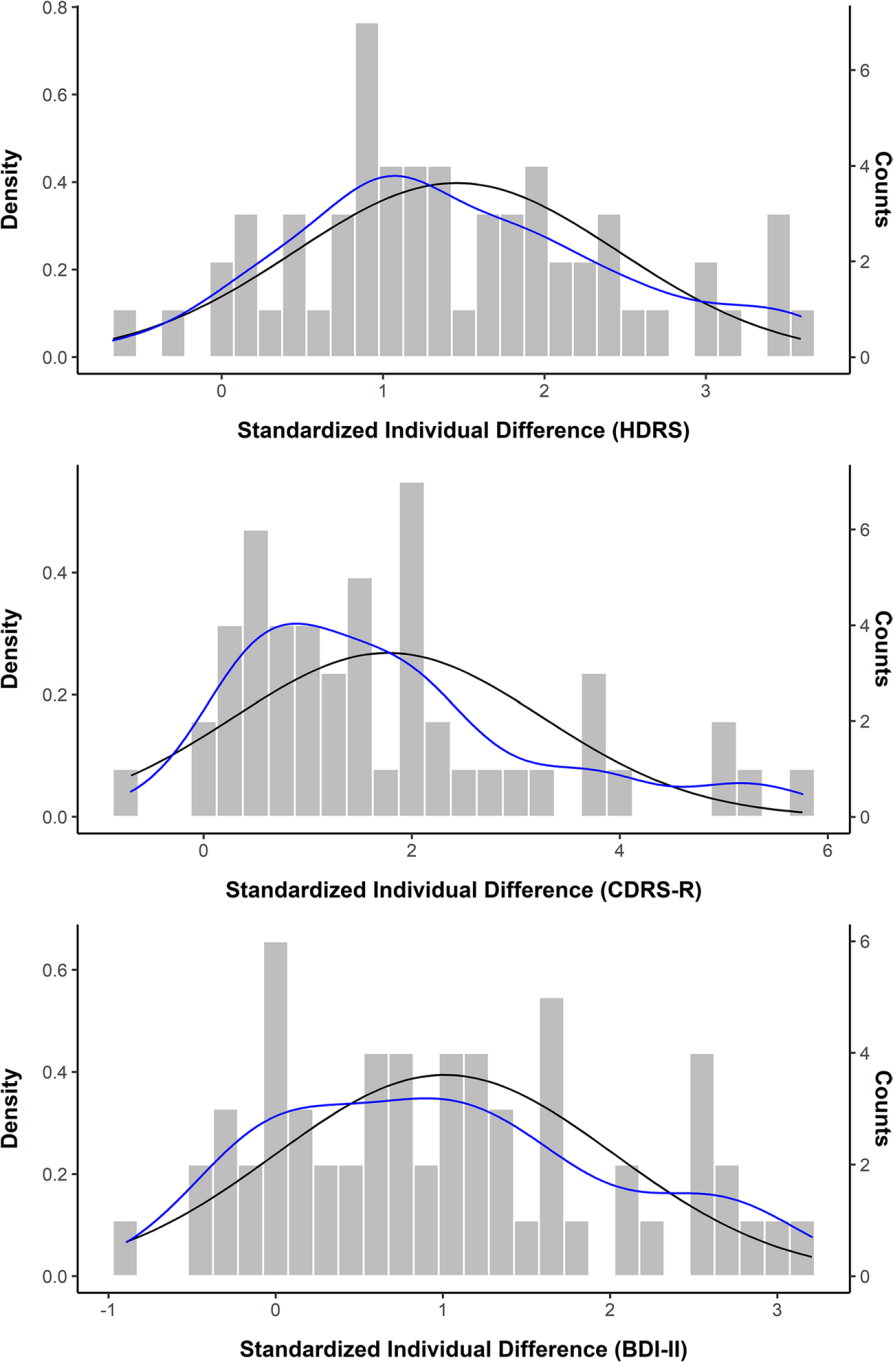
Histograms of standardized individual difference scores plotted separately for each outcome, including Gaussian (normal) curves (black) and individual (smoothed) density curves (blue). *BDI-II* Beck Depression Inventory-II; *CDRS-R* Children’s Depression Rating Scale; *HDRS* Hamilton Depression Rating Scale

Considering pre- to post-treatment change in depression scores, exploratory mixed-effects linear regression analysis, accounting for repeated measures within individuals clustered within trials, revealed a statistically significant pre- to post-treatment change considering each of the three depression scales examined (HDRS: *B* = – 8.72, *β* = – 1.23, *t*_59_ = – 10.62, *p* < 0.001; BDI-II: *B* = – 11.63, *β* = – 0.92, *t*_60_ = – 7.73, *p* < 0.001; CDRS-R: *B* = – 0.72, *β* = – 1.26, *t*_59_ = – 10.62, *p* < 0.001). Respective intra-class correlation coefficients (ICC) suggested that on average, about 26% of variance in outcome was due to clustering on the person-level (HDRS: 27%; CDRS: 15%; BDI-II: 37%). Trial level clustering on average accounted for another 26% of the variance (HDRS: 19%; CDRS: 36%; BDI-II: 24%). For the purpose of visualization, Standardized Individual Differences (*SID*; HDRS mean SID = 1.46, SD = 1.0, range: – 0.67 to 3.59; CDRS-R mean SID = 1.77; SD = 1.48, range: – 0.70 to 5.76; BDI-II mean SID = 1.01, SD = 1.01, range: – 0.89 to 3.21) were plotted using histograms (Fig. 5).

**Fig. 6:**
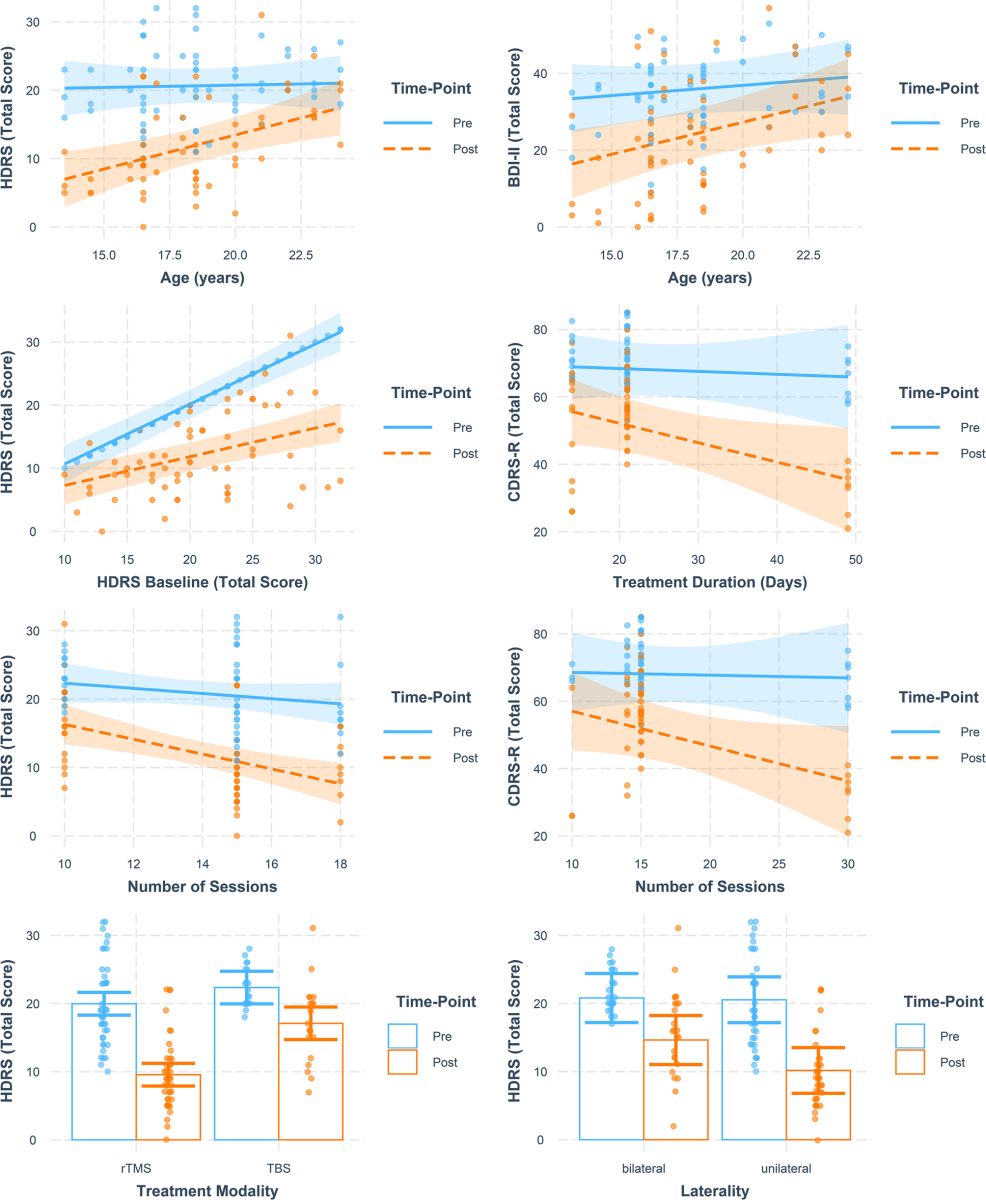
Interaction plots showing significant treatment–covariate interactions with patient- and treatment-level characteristics

Results from moderator analyses, that is, mixed-effects linear regression models examining treatment interactions with the patient- and treatment-level characteristics, while accounting for repeated measures and trial-level clustering, are summarized in Table S2. Based on available IPD from studies reporting on the HDRS, the patient-level characteristics age and severity of depression at pre-treatment (baseline) significantly moderated pre- to post-treatment change in depression severity. Specifically, younger age and higher severity of depression at baseline were associated with significantly higher reduction in HDRS scores between pre- and post-treatment measurements, as compared to higher-aged individuals and those with lower baseline depression scores. Considering treatment-level characteristics, pre- to post-treatment change in HDRS scores was significantly associated with ‘laterality’ (i.e., whether uni- or bilateral stimulation had been applied), the total number of stimulation sessions participants received, and the TMS modality applied (i.e., standard rTMS vs. TBS). With unilateral as compared to bilateral stimulation, a higher reduction in HDRS scores was observed, as with a higher number of sessions, and with standard rTMS compared to TBS. Of note, additionally controlling for the number of sessions when considering rTMS modality as a potential moderator did not significantly affect the respective results. Considering potential covariates of pre- to post-treatment change in CDRS-R scores, total number of sessions as well as treatment duration was identified as significant moderators. Both a higher number of sessions and a higher duration of treatment (higher number of days) were associated with significantly higher reductions in CDRS-R scores from pre- to post-treatment. Finally, considering BDI-II scores, pre- to post-treatment change was significantly associated with patient age, while the covariates ‘laterality’, number of TMS sessions, and TMS modality were trend significant. In addition, here, lower age was linked with significantly higher reductions in BDI-II scores at treatment termination. Interpretations of significant treatment-covariate interactions were examined visually by interaction-plots, as illustrated in Fig. 6.

#### Treatment response vs. non-response

**Fig. 7:**
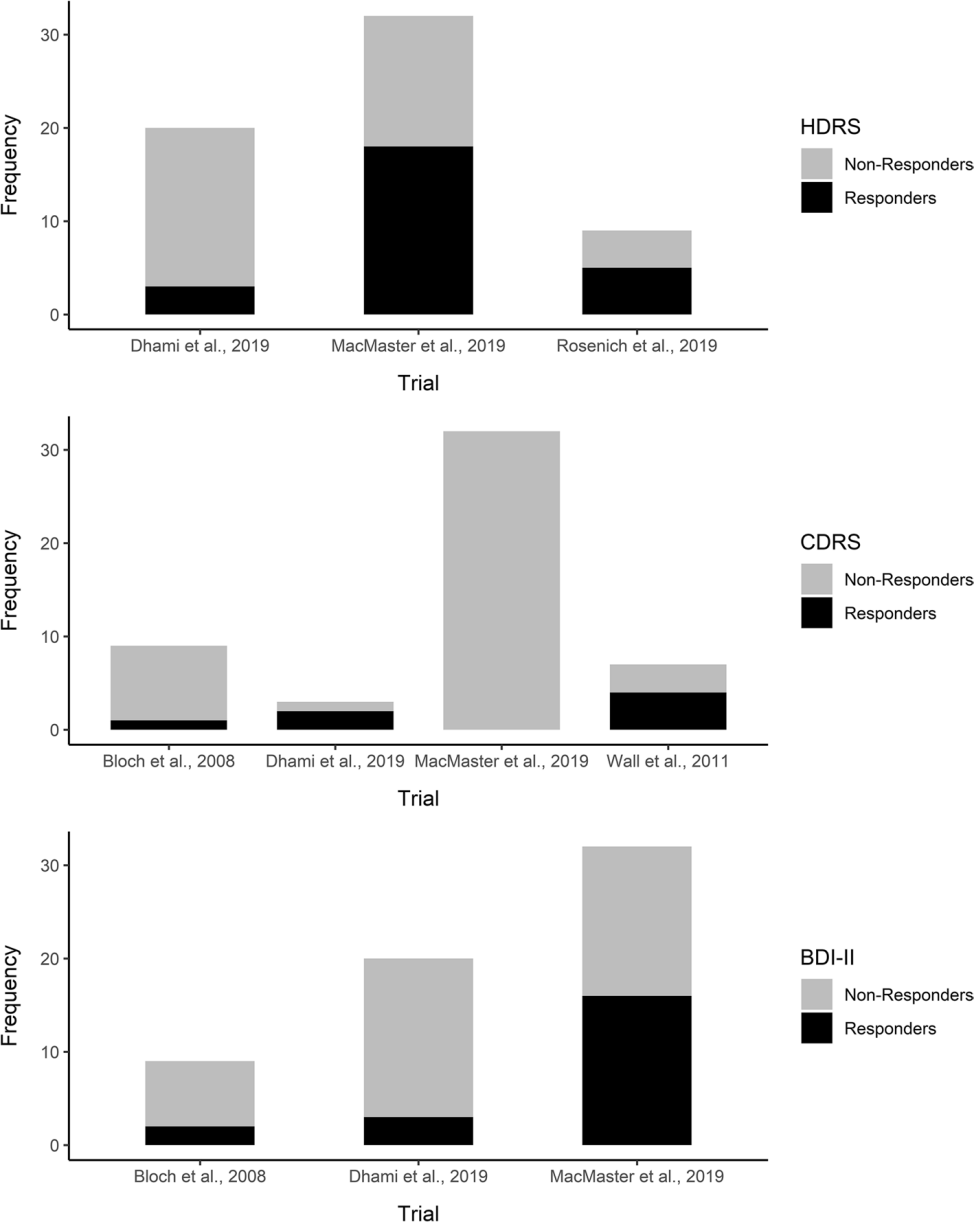
Histograms depicting treatment response vs. non-response by depression scale and trial

Considering exploratory IPD meta-analysis of treatment response vs. non-response, in total and on average, 30.26% of patients were considered as treatment responders (minimum 50% reduction from pre- to post-treatment). Based on HDRS scores, 42.62% of patients were considered as treatment responders, while 15% of variance in treatment response vs. non-response was explained by study-level clustering. Considering CDRS-R scores, only 13.73% of patients were treated as responders, and study-level clustering accounted for 61% of variance in treatment response. Regarding BDI-II scores, 34.43% of patients were considered treatment responders, with study-level clustering accounting for 11% of variance in treatment response. Rates of treatment response vs. non-response by outcome measure and trial are depicted in Fig. 7.

Results from mixed-effects logistic regression models accounting for clustering within trials and examining the potential influence of patient- and treatment-level characteristics on treatment response vs. non-response are summarized in Table S3. Based on available IPD and HDRS as well as the BDI-II scores, age was identified as significant patient-level covariate of treatment response, with a higher likelihood of treatment response linked with younger age. Considering treatment-level characteristics as potential covariates, ‘laterality’, the total number of stimulation sessions, and TMS modality, were identified as to significantly influence treatment response measured over both the HDRS and BDI-II scores. With unilateral as compared to bilateral stimulation, a higher likelihood of treatment response was observed, as with a higher number of treatment sessions, and standard rTMS compared to TBS. Of note, when considering CDRS-R scores, none of the covariates examined was found to significantly moderate treatment response.

## Discussion

The present systematic review and meta-analysis was conducted with the primary aims of quantifying efficacy of TMS in the treatment of adolescent depression and exploring respective patient- and trial-level moderators while considering the data currently available on a study (aggregated data) and patient-level (IPD), respectively. As a secondary aim, we also synthesized available data on rates of treatment response (aggregated data), and explored respective moderators (IPD).

First, meta-analysis of aggregated data suggested TMS to significantly reduce depression severity in adolescent patients, which was further in line with significant response rates. However, analyses of aggregated data also suggested potential biases, such as small-study effects. Indeed, the studies included were characterized by small sample sizes and large standard errors, respectively. Of note, all studies reported a statistically significant mean change in depression severity under active rTMS treatment from baseline to post-treatment, providing strong indications for potential publication bias—which was also suggested by visual inspection of funnel plots and formal testing of funnel plot asymmetry, respectively. Collectively, the present studies were also very heterogeneous with respect to dosing protocols for TMS. Of note, only two out of 10 studies included in the present synthesis applied a double-blind, randomized, and sham-controlled study design, and there have been considerable concerns about open-label trials to inherently inflate effect sizes and to be prone to several further biases, including regression to the mean, investigator biases, and, critically, confounding of active treatment with placebo effects [37, 83]. Sham stimulation in rTMS trials can be considered in analogy to pill placebo in pharmacological trials [37], and respective trials suggest larger response rates to pill placebo in adolescent as compared to adult depression [84, 85]—with reported rates of placebo response in adolescents ranging between 22 and 59% [86–93]. Considering rTMS treatment, there is only one large-scale randomized controlled study currently available that would inform on the response rate to sham stimulation in adolescent depression, suggesting a sham response rate of 36.4% [54]—which falls well within the range of response rates reported for pill placebo. As a comparison, meta-analytic studies suggest response rates to sham rTMS in adult patients with depression to be at around 10 or 11% [25, 94].

Based on IPD from five individual study samples, accounting for repeated measures within individuals clustered within trials, and considering three different depression scales (i.e., HDRS, CDRS-R, and BDI-II), we found treatment efficacy as well as response to be associated with certain patient and trial-level characteristics. Most consistently observed was the influence of patients’ age, with younger individuals exhibiting a higher reduction in depression scores as well as a higher likelihood of treatment response compared to individuals of older age. This finding somewhat aligns with existing meta-analytic evidence on TMS in adult patients suggesting young age to present a good prognostic factor [39], as well as with findings from an evidence synthesis of several short-term randomized controlled trials of antidepressants, reporting higher placebo response rates in younger as compared to older adolescents (after the exclusion of one large fluoxetine trial) [93]. Of note, presently, most studies included samples of older-aged adolescents (mean age of 17.45 ± 1.99 years), with only one study including relatively younger adolescents between 12 and 14 years. However, based on the present findings as well as growing evidence which points towards a favorable safety profile of TMS for the treatment of adolescent depression [52], future studies should also consider younger individuals with MDD. Furthermore, although only observed when considering HDRS but not CDRS-R or BDI-II scores, depression severity was a significant moderator of pre- to post-treatment change after TMS. This finding also aligns with a considerable amount of evidence in adults, suggesting TMS to be particularly valuable in severe cases of adult MDD, and in patients with TRD [25, 27, 95]. TMS applied to the DLPFC in adolescent MDD might reverse some of the aberrant functional connectivity between prefrontal and subcortical regions, which, during the period of adolescence that is characterized by peak PFC plasticity, might result in long-term clinical improvement, especially in severe cases of depression [37]. Considering the present variability of results based on the outcome measure considered, several potential explanations for this finding exist. First, the present findings based on IPD analyses in general might simply present spurious associations that might have occurred based on a multiple testing situation, and future studies with pre-planned hypotheses testing will be needed to confirm the observed associations. Furthermore, there might be poor concordance between different instruments, i.e., clinician-rated (such as the HDRS or MADRS) and patient-reported (BDI-II) outcome measures of symptoms of depression [96]. Furthermore, specifically considering the CDRS-R, which currently presents the most commonly used scale in adolescent depression research, existing studies on the psychometric properties of this instrument when used in adolescents (but which was originally developed for use in children aged 6–12 years) are of low methodological quality, and thus it currently remains unclear whether the CDRS-R appropriately measures depressive symptom severity in adolescent MDD [97]. Finally, differences in IPD analysis results considering different measures might also go back to underlying sample characteristics, which presently has not been explored further but should be considered in future antidepressant trial. Besides patient factors, several trial-specific, TMS-related factors were identified to significantly moderate treatment efficacy as well as the likelihood of treatment response. These included the laterality of stimulation, the specific TMS modality applied, treatment duration, and the number of stimulation sessions applied, respectively. Concerning the latter, greater efficacy of TMS has also been previously reported for protocols applying a higher number of stimulation sessions, as well as a greater number of pulses per session [98–100]. In the five studies included in IPD meta-analysis, the number of sessions ranged from 10 to 30 sessions, with a treatment duration of 14–42 days. It has been previously suggested that increasing the number of sessions per day from one to multiple might increase efficiency [37]. Yet, as presently observed, a longer treatment duration might also increase efficacy (although potentially confounded by the number of sessions applied). Consequently, future studies should investigate the relative importance of the number of TMS sessions and overall treatment duration considering treatment efficacy to determine a session/duration ratio that optimizes stimulation protocols for both efficacy and efficiency. Concerning laterality of stimulation, the present finding of greater efficacy observed with unilateral compared to bilateral stimulation is in line with results from a double-blind, randomized, and sham-controlled study in adults with TRD [101]. In the respective study, only unilateral stimulation was significantly more effective compared to sham stimulation at treatment termination and was correlated with a higher percentage of patients who showed remission. Further, unilateral but not bilateral TMS showed higher antidepressant efficacy compared to sham stimulation in the respective study. However, these and the present findings are somewhat contradictory to existing meta-analytic evidence, suggesting that bilateral stimulation might not be statistically significantly different from unilateral stimulation in adult MDD [102–104]. Given potential neurodevelopmentally driven differences between the pathophysiology of MDD in adolescents compared to adults, it is important that future studies further investigate whether unilateral compared to bilateral stimulation might be differentially effective in adolescent MDD. Similar, standard rTMS compared to TBS was associated with greater treatment response. Of note, only one of the included studies used TBS. Primary studies addressing the comparative efficacy of rTMS versus TBS are warranted.

The present results must be interpreted within the constraints of considerable limitations inherent to this evidence synthesis. First, given the current lack of randomized controlled trials considering TMS treatment in minors with depression, we failed to conduct a synthesis of results from rigorous randomized controlled trials comparing active rTMS treatment with sham stimulation. Instead, we conducted a quantitative synthesis of results from mainly uncontrolled studies. While non-randomized studies are increasingly recognized as a potential source of insights into real-world performance of novel therapeutics, and thus are certainly of value for healthcare decision making especially in the case of novel and innovative treatments [105], high-quality, randomized controlled trials unequivocally provide the most reliable evidence on the relative efficacy and safety of medical interventions. The present results must therefore be considered highly cautiously. As a further critical limitation, individual-patient data were retrieved only for half (i.e., five out of ten) of the studies that were included in the present meta-analysis, and most critically, we failed to include IPD data from randomized controlled trials—which certainly would have significantly improved the quality of the data included and allowed us to also consider effects of sham stimulation in IPD meta-analysis. Barriers to obtaining IPD on the one hand were encountered due to a lack of responsiveness of the corresponding authors even to repeated data requests. Furthermore, barriers were also encountered in the form of data sharing policies of study sponsors from the industry. Either problem is strongly impeding on the quality of meta-science, and endeavors to further ameliorate and facilitate practices of data sharing are warranted (for a scoping review and practical guide on the matter, we refer the reader to, e.g., [106]).

## Conclusion

The present meta-analysis is the first to synthesize existing evidence, consisting of mainly uncontrolled trials, for the use of TMS in adolescents with MDD. We found that TMS might be an efficient treatment for adolescents with MDD, in particular for those of younger age. Several treatment modality settings were identified that significantly influence treatment outcomes. Given the methodological limitations of primary studies included in the present meta-analysis, these should be interpreted with caution but—in the current absence of better evidence—may inform clinical practice in the application of TMS in youth with MDD.

## Supplementary Information

Below is the link to the electronic supplementary material.

Supplementary file1 (DOCX 45 kb)
