# Supplementary material for: Transcranial magnetic stimulation in the treatment of adolescent depression: a systematic review and meta-analysis of aggregated and individual-patient data from uncontrolled studies
Source: Eur Child Adolesc Psychiatry. 2022 Jun 24;31(10):1501–25. doi: 10.1007/s00787-022-02021-7 (PMC9532325; doi:10.1007/s00787-022-02021-7)
Supplement: Supplementary file 1 — Supplementary file1 (DOCX 45 kb) [file 787_2022_2021_MOESM1_ESM.docx]

**Supplementary Materials**

*Table S1.* Bivariate correlation coefficients between pre- and post-treatment depression scores measured over three different depressions scales (HDRS, BDI-II, and CDRS-R), based on IPD.

|  | Baseline HDRS | Baseline  BDI-II | Baseline  CDRS-R | Postline  HDRS | Postline  BDI-II | Postline  CDRS-R |
| --- | --- | --- | --- | --- | --- | --- |
| Baseline HDRS | - | - | - | - | - | - |
| Baseline  BDI-II | .50  <0.001  52 | - | - | - | - | - |
| Baseline  CDRS-R | .57 <0.001  35 | .46  0.002  44 | - | - | - | - |
| Postline  HDRS | .43  <.001  61 | .61  <0.001  52 | .35  0.037  35 | - | - | - |
| Postline  BDI-II | .38  0.009  52 | .59  <0.001  52 | .35  0.021  44 | .85  <.001  52 | - | - |
| Postline  CDRS-R | .23  .175  35 | .03  .862  44 | .50  <.001  51 | .58  <0.001  35 | .57  <0.001  35 | - |

*Note.*

*Table S2*. Pre-to-post treatment change in interaction with patient- and treatment level characteristics (reported separately for HDRS, CDRS, and BDI-II).

| Outcome |  |  |  |  |  |  |  |  |  | |  |
| --- | --- | --- | --- | --- | --- | --- | --- | --- | --- | --- | --- |
| HDRS | Predictors | Factor Level(s) | *B* | SE | *t* | *DF* | *p* |  | ICC: Individual-level | ICC: Trial-level | |
|  | Treatment (T) | Binary (Pre-Treatment; Post-Treatment) | -8.72 | .82 | -10.62 | 60 | <0.001 |  | 27% | 19% | |
|  | Treatment (T) | Binary (Pre-Treatment; Post-Treatment) | -25.85 | 5.26 | -4.91 | 59 |  |  |  |  | |
|  | T x Age |  | .93 | .28 | 3.29 | 59 | 0.002 |  |  |  | |
|  | Age | Continuous (Years) | .07 | .31 | .22 | 48 |  |  |  |  | |
|  | Treatment (T) | Binary (Pre-Treatment; Post-Treatment) | -9.71 | 1.15 | -8.47 | 59 |  |  |  |  | |
|  | T x Sex |  | 2.01 | 1.64 | 1.23 | 59 | 0.224 |  |  |  | |
|  | Sex | Binary (Male; Female) | -1.41 | 1.43 | -.98 | 103 |  |  |  |  | |
|  | Treatment (T) | Binary (Pre-Treatment; Post-Treatment) | 1.58 | 2.77 | .57 | 116 |  |  |  |  | |
|  | T x Depression severity |  | -.50 | .13 | -3.84 | 116 | <0.001 |  |  |  | |
|  | Depression severity | Continuous (Baseline Total Scores) | .95 | .09 | -3.84 | 116 |  |  |  |  | |
|  | Treatment (T) | Binary (Pre-Treatment; Post-Treatment) | -6.17 | 1.25 | -4.94 | 59 |  |  |  |  | |
|  | T x Laterality |  | -4.21 | 1.60 | -2.63 | 59 | 0.011 |  |  |  | |
|  | Laterality | Binary (Bilateral; Unilateral) |  |  |  |  |  |  |  |  | |
|  | Treatment (T) | Binary (Pre-Treatment; Post-Treatment) | 1.03 | 3.89 | .26 | 59 |  |  |  |  | |
|  | T x Number of Sessions |  | -.71 | .28 | -2.56 | 59 | 0.013 |  |  |  | |
|  | Number of Sessions | Continuous (Count) | -.38 | .30 | -1.27 | 2 |  |  |  |  | |
|  | Treatment (T) | Binary (Pre-Treatment; Post-Treatment) | -6.52 | 2.15 | -3.03 | 59 |  |  |  |  | |
|  | T x Treatment Duration |  | -.10 | .09 | -1.11 | 59 | 0.273 |  |  |  | |
|  | Treatment Duration | Continuous (Days) | -.09 | .15 | -.62 | 2 |  |  |  |  | |
|  | Treatment (T) | Binary (Pre-Treatment; Post-Treatment) | -12.25 | 7.65 | -1.60 | 49 | .116 |  |  |  | |
|  | T x Modality |  | 5.13 | 1.69 | 3.03 | 59 | .004 |  |  |  | |
|  | Modality | Binary (standard rTMS; TBS) | 2.37 | 1.47 | 1.61 | 103 |  |  |  |  | |
| CDRS-R | Predictors | Factor Level(s) | *B* | SE | *t* | *DF* | *p* |  | ICC: Individual-level | ICC: Trial-level | |
|  | Treatment (T) | Binary (Pre-Treatment; Post-Treatment) | -17.80 | 1.83 | -9.74 | 50 | <0.001 |  | 15% | 36% | |
|  | Treatment (T) | Binary (Pre-Treatment; Post-Treatment) | -9.02 | 21.96 | -.41 | 49 |  |  |  |  | |
|  | T x Age |  | -.52 | 1.30 | -.40 | 49 | 0.690 |  |  |  | |
|  | Age | Continuous (Years) | 1.08 | 1.05 | 1.03 | 89 |  |  |  |  | |
|  | Treatment (T) | Binary (Pre-Treatment; Post-Treatment) | -16.64 | 2.80 | -5.93 | 49 |  |  |  |  | |
|  | T x Sex |  | -2.05 | 3.72 | -.55 | 49 | 0.583 |  |  |  | |
|  | Sex | Binary (Male; Female) | 1.97 | 3.11 | .63 | 88 |  |  |  |  | |
|  | Treatment (T) | Binary (Pre-Treatment; Post-Treatment) | -9.58 | 4.65 | -.65 | 95 |  |  |  |  | |
|  | T x Depression severity |  | -.11 | .20 | -.57 | 95 | 0.573 |  |  |  | |
|  | Depression severity | Continuous (Baseline Total Scores) | .86 | .15 | 5.83 | 96 |  |  |  |  | |
|  | Treatment (T) | Binary (Pre-Treatment; Post-Treatment) | -29.33 | 7.42 | -3.95 | 49 |  |  |  |  | |
|  | T x Laterality |  | 12.25 | 7.65 | 1.60 | 35 | 0.116 |  |  |  | |
|  | Laterality | Binary (Bilateral; Unilateral) | .52 | 11.93 | .04 | 3 |  |  |  |  | |
|  | Treatment (T) | Binary (Pre-Treatment; Post-Treatment) | -1.95 | 5.39 | -.36 | 49 |  |  |  |  | |
|  | T x Number of Sessions |  | -.96 | .31 | -3.10 | 49 | 0.003 |  |  |  | |
|  | Number of Sessions | Continuous (Count) | -.08 | .31 | -3.10 | 2 |  |  |  |  | |
|  | Treatment (T) | Binary (Pre-Treatment; Post-Treatment) | -6.41 | 4.03 | -1.59 | 49 |  |  |  |  | |
|  | T x Treatment Duration |  | -.49 | .16 | -3.11 | 49 | 0.003 |  |  |  | |
|  | Treatment Duration | Continuous (Days) | -.08 | -28 | 2.28 | 2 |  |  |  |  | |
|  | Treatment (T) | Binary (Pre-Treatment; Post-Treatment) | -17.08 | 1.86 | -9.20 | 49 |  |  |  |  | |
|  | T x Modality |  | -.52 | 11.93 | -.04 | 49 | .116 |  |  |  | |
|  | Modality | Binary (standard rTMS; TBS) | -12.25 | 7.65 | -1.60 | 3 |  |  |  |  | |
| BDI-II | Predictors | Factor Level(s) | *B* | SE | *t* | *DF* | *p* |  | ICC: Individual-level | ICC: Trial-level | |
|  | Treatment (T) |  | -11.63 | 1.51 | -7.73 | 60 | <0.001 |  | 34% | 24% | |
|  | Treatment (T) |  | -32.43 | 10.02 | -3.24 | 59 |  |  |  |  | |
|  | T x Age |  | 1.14 | .54 | 2.10 | 59 | 0.040 |  |  |  | |
|  | Age | Continuous (Years) | .53 | .65 | 2.10 | 65 |  |  |  |  | |
|  | Treatment (T) | Binary (Pre-Treatment; Post-Treatment) | -11.62 | 2.20 | -5.28 | 59 |  |  |  |  | |
|  | T x Sex |  | -.03 | 3.04 | -.009 | 59 | 0.993 |  |  |  | |
|  | Sex | Binary (Male; Female) | 3.52 | 2.88 | 1.22 | 97 |  |  |  |  | |
|  | Treatment | Binary (Pre-Treatment; Post-Treatment) | -6.89 | 5.39 | -1.28 | 116 |  |  |  |  | |
|  | T x Depression severity |  | -.94 | .11 | -.92 | 116 | 0.361 |  |  |  | |
|  | Depression severity | Continuous (Baseline Total Scores) | .94 | .11 | 8.47 | 114 |  |  |  |  | |
|  | Treatment (T) | Binary (Pre-Treatment; Post-Treatment) | -7.80 | 2.58 | -3.02 | 59 |  |  |  |  | |
|  | T x Laterality |  | -5.70 | 3.15 | -1.81 | 59 | 0.075 |  |  |  | |
|  | Laterality | Binary (Bilateral; Unilateral) | -3.60 | 9.44 | -.38 | 1 |  |  |  |  | |
|  | Treatment (T) | Binary (Pre-Treatment; Post-Treatment) | 4.32 | 8.72 | .50 | 59 |  |  |  |  | |
|  | T x Number of Sessions |  | -1.22 | .62 | -1.96 | 50 | 0.056 |  |  |  | |
|  | Number of Sessions | Continuous (Count) | -1.16 | 1.74 | .94 | 1 |  |  |  |  | |
|  | Treatment (T) | Binary (Pre-Treatment; Post-Treatment) | .43 | 7.66 | .06 | 59 |  |  |  |  | |
|  | T x Treatment Duration |  | -.68 | .43 | -1.61 | 59 | 0.114 |  |  |  | |
|  | Treatment Duration | Continuous (Days) | -1.28 | .40 | -3.17 | 98 |  |  |  |  | |
|  | Treatment (T) | Binary (Pre-Treatment; Post-Treatment) | -13.51 | 1.80 | -7.49 | 59 |  |  |  |  | |
|  | T x Modality |  | 5.71 | 3.15 | 1.81 | 59 | .075 |  |  |  | |
|  | Modality | Binary (standard rTMS; TBS) | 3.60 | 9.44 | .38 | 1 |  |  |  |  | |

*Note*. BDI-II = Beck Depression Inventory-II; CDRS-R = Children’s Depression Rating Scale‒Revised; HDRS = Hamilton Depression Rating Scale.

*Table S3*. Patient- and treatment level characteristics as predictors of treatment response vs. non-response, reported separately for HDRS (*N* = 61), CDRS (*N* = 51), and BDI-II (*N* = 61).

| Treatment Response | |  |  |  |  |  |  |  |  |
| --- | --- | --- | --- | --- | --- | --- | --- | --- | --- |
| HDRS | Predictors | Factor Level(s) | *B* | SE | z | *p* |  | *DF* resid. | ICC: Trial-level |
|  |  |  |  |  |  |  |  |  | 15% |
|  | Age | Continuous (Years) | -.38 | .13 | -2.97 | .003 |  | 58 |  |
|  | Sex | Binary (Male; Female) | -.84 | .57 | -1.48 | .139 |  | 58 |  |
|  | Depression severity | Continuous (Baseline Total Scores) | .07 | .05 | 1.36 | .174 |  | 58 |  |
|  | Laterality | Binary (Bilateral; Unilateral) | 1.61 | .60 | 2.67 | .008 |  | 58 |  |
|  | Number of Sessions | Continuous (Count) | .29 | .11 | 2.68 | .007 |  | 34 |  |
|  | Treatment Duration | Continuous (Days) | .05 | .04 | 1.30 | .196 |  | 58 |  |
|  | T x Modality | Binary (standard rTMS; TBS) | -1.98 | .70 | -2.84 | .005 |  | 58 |  |
|  |  |  |  |  |  |  |  |  |  |
| CDRS-R | Predictors | Factor Level(s) | *B* | SE | z | *p* |  | *DF* resid | ICC: Trial-level |
|  |  |  |  |  |  |  |  |  | 61% |
|  | Age | Continuous (Years) | .07 | .003 | .603 | .546 |  | 48 |  |
|  | Sex | Binary (Male; Female) | -.04 | 1.52 | -.02 | .981 |  | 48 |  |
|  | Depression severity | Continuous (Baseline Total Scores) | .04 | .10 | .424 | .672 |  | 48 |  |
|  | Laterality | Binary (Bilateral; Unilateral) | -3.17 | 2.58 | -1.23 | .219 |  | 48 |  |
|  | Number of Sessions | Continuous (Count) | .08 | .19 | .43 | .671 |  | 45 |  |
|  | Treatment Duration | Continuous (Days) | .06 | .07 | .78 | .433 |  | 39 |  |
|  | T x Modality | Binary (standard rTMS; TBS) | 3.17 | 2.58 | 1.23 | .219 |  | 58 |  |
|  |  |  |  |  |  |  |  |  |  |
| BDI-II | Predictors | Factor Level(s) | *B* | SE | *z* | *p* |  | *DF* resid. | ICC: Trial-level |
|  |  |  |  |  |  |  |  |  | 11% |
|  | Age | Continuous (Years) | -.43 | .15 | -2.80 | .005 |  | 58 |  |
|  | Sex | Binary (Male; Female) | .09 | .57 | .158 | .875 |  | 58 |  |
|  | Depression severity | Continuous (Baseline Total Scores) | .00 | .03 | .104 | .918 |  | 58 |  |
|  | Laterality | Binary (Bilateral; Unilateral) | 1.49 | .70 | 2.13 | .034 |  | 58 |  |
|  | Number of Sessions | Continuous (Count) | .36 | .14 | 2.41 | .016 |  | 38 |  |
|  | Treatment Duration | Continuous (Days) | .22 | .09 | 2.59 | .010 |  | 49 |  |
|  | T x Modality | Binary (standard rTMS; TBS) | -1.49 | .70 | -2.13 | .034 |  | 48 |  |

*Note*. BDI-II = Beck Depression Inventory-II; CDRS-R = Children’s Depression Rating Scale‒Revised; HDRS = Hamilton Depression Rating Scale.
